# Closed-loop atomic force microscopy-infrared spectroscopic imaging for nanoscale molecular characterization

**DOI:** 10.1038/s41467-020-17043-5

**Published:** 2020-06-26

**Authors:** Seth Kenkel, Shachi Mittal, Rohit Bhargava

**Affiliations:** 1 Beckman Institute for Advanced Science and Technology, University of Illinois at Urbana Champaign, Urbana, IL 61801 USA; 2 Department of Mechanical Engineering, University of Illinois at Urbana Champaign, Urbana, IL 61801 USA; 3 Cancer Center at Illinois and the Departments Chemical and Biomolecular Engineering, Bioengineering, Electrical and Computer Engineering, and Chemistry, University of Illinois at Urbana-Champaign, Urbana, IL 61801 USA

**Keywords:** Infrared spectroscopy, Characterization and analytical techniques

## Abstract

Atomic force microscopy-infrared (AFM-IR) spectroscopic imaging offers non-perturbative, molecular contrast for nanoscale characterization. The need to mitigate measurement artifacts and enhance sensitivity, however, requires narrowly-defined and strict sample preparation protocols. This limits reliable and facile characterization; for example, when using common substrates such as Silicon or glass. Here, we demonstrate a closed-loop (CL) piezo controller design for responsivity-corrected AFM-IR imaging. Instead of the usual mode of recording cantilever deflection driven by sample expansion, the principle of our approach is to maintain a zero amplitude harmonic cantilever deflection by CL control of a subsample piezo. We show that the piezo voltage used to maintain a null deflection provides a reliable measure of the local IR absorption with significantly reduced noise. A complete analytical description of the CL operation and characterization of the controller for achieving robust performance are presented. Accurate measurement of IR absorption of nanothin PMMA films on glass and Silicon validates the robust capability of CL AFM-IR in routine mapping of nanoscale molecular information.

## Abstract (web summary)

Atomic force microscopy-infrared (AFM-IR) spectroscopic imaging techniques offer a non-perturbative, molecular contrast for characterization of nanomaterials; however, data are often complicated by the measurement apparatus, sample preparation conditions and low signal-to-noise ratio. Here, the authors demonstrate a closed-loop controlled AFM-IR instrument design to address measurement artifacts and reduce noise up to 5x compared to previous methods.

## Introduction

Realizing the extraordinary potential of nanoscale materials^1–5^ for diverse applications such as high frequency nanoelectronics^6,7^, NEMS and MEMS devices^8^, and photonics^9^ requires reliable characterization tools. This need has driven significant advances in metrology capabilities using now well-established tools such as atomic force microscopy (AFM)^10^ and transmission electron microscopy (TEM)^11^ for visualizing both the physical structure and chemical composition of nanomaterials with sufficient spatial and molecular resolution. However, mapping the chemical composition of these materials remains a challenge that requires labeling^12^ or spectroscopic techniques. Infrared (IR) and Raman spectroscopy have been used extensively to study nanostructured materials^6^ by detecting molecular composition^13^ in bulk; however, the capabilities of far-field techniques are not suited for visualizing nanoscale materials.

Near-field spectroscopic methods are needed, which typically couple the contrast of vibrational spectroscopy with AFM^14–22^ mapping. However, near-field approaches are prone to artifacts whose understanding is critical for optimizing analytical measurements^23,24^ but as yet incomplete. For example, IR scattering scanning near-field optical microscopy (IR s-SNOM) and tip-enhanced Raman spectroscopy (TERS) rely on specially designed probes^25^ for enhancing the near-field signal as well as complex, tip-specific models^16,26^ for interpreting the recorded chemical data. While tip models offer a much-needed but approximate understanding of the measured contrast, quantification of results is complicated by the difficult to predict tip-sample interactions^27^. Similarly, while enhancement of the near field signal is necessary for measurements to be feasible, it can result in confounding factors that are difficult to control or mitigate experimentally. Small changes in experimental parameters can lead to large changes in recorded signals, amplified noise and inconsistent (less reproducible) measurements often imposing restrictions on sample preparation. This lack of consistency derives from a lack of understanding of the dominant contributions to the recorded signal that does not allow for easy optimization. For example, innovative new techniques such as photo-induced force microscopy (PiFM) have great potential^20^, but the recorded signal reportedly arises from a mixture of optical forces^20,28^, tip-enhanced^29^ and direct^30^ thermal expansion and photoacoustic effects^30^. Here we report a near-field spectroscopic method that ensures both robust and routine analytical measurements enabled by a thorough fundamental understanding, evaluation of relative contributions of nanoscale processes and systematic optimization of data recording.

We focused on AFM-IR spectroscopic imaging as it involves a relatively simple mechanism of detecting the molecular absorption-induced thermal expansion of the sample, does not need the aid of a theoretical model for interpreting data, provides chemical contrast without labels and produces signal shown to be correlated to far-field absorption spectra^31,32^. Despite these advantages, AFM-IR has a number of limitations that remain unsolved. Current state-of-the-art resonance-enhanced methods have shown monolayer sensitivity in detection; however, these studies rely on the signal enhancement of gold or polymer coated substrates.^20,33–35^ In addition, although the recorded signal (to first approximation) is proportional to far-field IR absorption, recent reports demonstrate the measured AFM-IR contrast is typically composed of contributions from chemical composition as well as mechanical features that arise from cantilever responsivity variations.^36,37^ Correcting for the cantilever responsivity using a subsample piezo expansion has been shown to improve chemical accuracy of the recorded contrast, but offers little improvement in sensitivity compared to the previous state-of-the-art^20,33,34^. Thus, despite the relative simplicity and the potential of AFM-IR, it remains mired in many of the same trade-offs as other near-field approaches. Enhancement, need for narrowly defined sample preparation, convolution with confounding factors and low chemical sensitivity (especially compared to state-of-the-art IR microscopy^38^) together restrict the analytical potential and fidelity of current AFM-IR methods to study molecular properties of nanoscale materials on substrates of importance to the nanotechnology community such as Silicon or glass.

Here, we develop the theory and demonstrate implementation of a robust closed-loop (CL) responsivity-corrected AFM-IR measurement capability. Our design is geared to be universally applicable to minimizing measurement noise in AFM-IR, thereby enabling accurate, high sensitivity compositional mapping at the nanoscale without the need for overly restrictive sample preparation methods or need for specialized substrates. In contrast to the current practice of recording signal arising from cantilever deflection, the principle of our strategy is to modulate and record a harmonic voltage applied to a subsample mechanical actuator (piezo) to maintain a near-zero amplitude harmonic cantilever deflection voltage by real-time feedback control. This “closed-loop” method of operation can greatly reduce susceptibility of the recorded chemical signal to both spatial and time-varying changes in cantilever resonance, thereby simultaneously improving the chemical accuracy while reducing noise. Further, the strategy of maintaining a null deflection obviates issues of detector saturation to enable the simultaneous use of high laser power and cantilever resonance amplification. Together, the CL approach seeks to make AFM-IR a more sensitivity, accurate and easier to use technique. To demonstrate, we first describe the AFM-IR signals theoretically then provide a complete design, analysis and characterization of the controls for robust, high quality measurements. We then demonstrate improvements of the developed instrumentation relative to prior state-of-the-art methods by mapping the IR absorption of nanoscale-thick PMMA films on glass and Silicon eliminating the need for restrictive sample preparation on gold or polymer coated substrates^20,33–35^.

## Results

### Concept for closed-loop AFM-IR

The foundation of our approach is shaped by recent progress in understanding of the recorded AFM-IR signal. The signal recorded in conventional AFM-IR measurements is the amplitude of the harmonic cantilever deflection driven by absorption-induced sample expansion. To first approximation, this signal is proportional to the local IR absorption of the sample near the cantilever tip^21^; however, probe-sample coupling^36^ results in non-molecular contributions to the signal. The source of this non-chemical contrast can be described as variations in the cantilever’s responsivity^37^ which relates any out-of-plane, free-surface sample expansion to the recorded cantilever deflection voltage. Measuring cantilever deflection driven by a subsample piezo to estimate responsivity can help correct for non-chemical contrast (hereafter referred to as open-loop (OL) responsivity corrected AFM-IR or just OL), improving on post-processing methods such as IR peak ratios^36^, experimental techniques such as resonance tracking^39^ (hereafter referred to as resonance enhanced (RE) AFM-IR or just RE) or conventional AFM-IR cantilever ring down measurements (hereafter referred to as photo thermal induced resonance or PTIR). While interlaced piezo and QCL signal acquisitions can be used to mitigate responsivity contributions, it is not possible to demodulate both signals in real time. Due to low signal and repeatability challenges associated with imaging nanoscale materials, factors such as noise and sample drift that are typical of AFM measurements can significantly influence ratio data of fine sample features. Additionally, measuring two signals for a correction can only possibly decrease the SNR from the levels of either one. Thus, while this approach shows utility for responsivity correction of coarser features, smoothing fine features and no native enhancement of the signal result in a trade-off between accuracy and sensitivity in nanoscale measurements. Given the rigorous analytical approach to responsivity correction developed, we follow this path to seek an AFM-IR design that obviates this trade-off.

### CL piezo controller design

The CL formulation proposed here is just such a design. The first step to implementing it consists of describing the process mathematically. To begin this description, consider the governing behavior of a cantilever

1 $$D\left[ n \right] = H_c\left[ n \right]\left( {{\it{\epsilon }}_1\left[ n \right] + H_pv_2\left[ n \right]} \right).$$

Here we have related the complex-valued modulation amplitude of the harmonic signals sampled in discrete time *n* where *D* is the cantilever deflection, *H*_*c*_ is the cantilever transfer function (or responsivity), ${\it{\epsilon }}_1$ is the IR photo-induced, out-of-plane, free surface expansion amplitude and $v_2$ is the piezo modulation voltage. The resulting expansion amplitude of the piezo ${\it{\epsilon }}_2 = H_pv_2$ is mathematically equivalent to an amplifier bias (via interference of the two harmonic expansion signals ${\it{\epsilon }}_1$ and ${\it{\epsilon }}_2$). This insight allows us to hypothesize that a bias can be applied to the IR expansion to maintain a constant deflection signal. Specifically, holding the deflection to zero provides a piezo voltage that is proportional to the photo-induced sample expansion. Hence, our proposed approach of maintaining zero deflection through control of the piezo voltage offers a alternative way to record a signal (piezo voltage) which converges to the AFM-IR molecular expansion signal in real time, free from both spatial and temporal variations in cantilever responsivity (hereafter referred to as CL responsivity corrected AFM-IR or just CL).

Although the transient photothermal sample response has been reported as fast as 10 ns^40^, this does not fundamentally restrict the temporal resolution requirements of the proposed CL controller. The CL design incorporates heterodyne detection of a quasi-steady state harmonic signal induced by a pulsed IR laser. While a fast thermal-mechanical sample response requires high temporal resolution in time-domain measurements, the quasi-steady harmonic signal obviates the need for high-bandwidth measurement. The only transient effect which needs to be accounted for is the AFM scan speed which is quite slow relative to the transient response of the cantilever probe. For a noiseless, artifact-free system, the CL approach can perfectly nullify the harmonic expansion signal regardless of the transient thermal response of the sample.

**Fig. 1:**
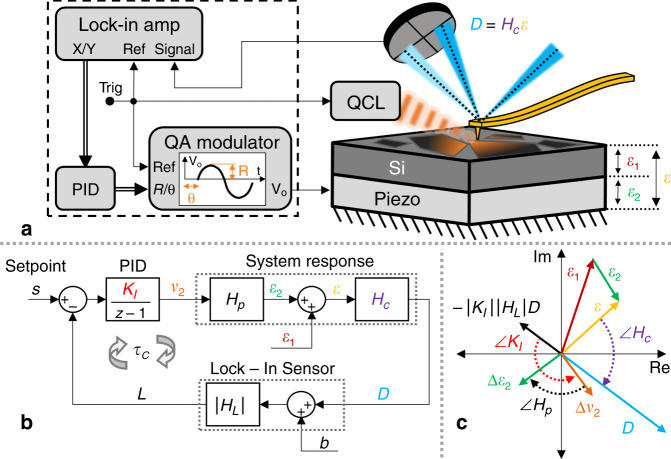
Closed-loop AFM-IR piezo controller schematic. **a** AFM-IR schematic (right) with QCL and piezo induced harmonic expansion amplitudes $\epsilon _1$ and $\epsilon _2$ respectively. The total expansion amplitude $\epsilon$ is detected via the cantilever’s deflection voltage which is demodulated using a dual lock-in amplifier (left). The x and y demodulated signals are processed via a discrete-time PID controller, the output of which is feed to a Quadrature Amplitude Modulator (QAM). The harmonic voltage output of the QAM is used to drive the piezo expansion. **b** Discrete-time, closed-loop piezo controller diagram depicting the AFM-IR system response, lock-in sensor and PID controls. A discrete-time integral controller with adjustable gain *K*_*I*_ allows for sufficient control of the piezo modulation voltage *v*_2_ in order to maintain a nominally zero amplitude harmonic deflection voltage. **c** Phasor diagram of the complex-valued signals showing how the change in the piezo expansion amplitude $\Delta \epsilon _2$ at each iteration will reduce the total expansion amplitude $\epsilon$. For stable performance, the total expansion (and deflection) converge to zero. The integral gain *K*_*I*_ can be adjusted in real time to compensate for variable cantilever responsivity *H*_*c*_ to ensure stable and optimal controller performance.

Figure 1a depicts a piezo-controller design based on the CL concept. The operation is similar to conventional AFM-IR, wherein a pulsed, IR source is weakly focused to the sample under the probe tip and the resulting photo-induced thermal expansion drives a harmonic cantilever oscillation which is recorded using the AFM electronics (such as a quad diode). This signal is demodulated using a dual Lock-in amplifier to extract both the X and Y modulation voltages. The relation between the cantilever deflection modulation amplitude and the recorded lock-in signal can be defined as follows

2 $$L\left[ n \right] = \left| {H_L} \right|\left( {D\left[ n \right] + b} \right).$$

Here, *L*[*n*] is the demodulated X and Y lock-in voltages (described mathematically as a complex-valued signal), |*H*_*L*_| and *b* are the lock-in gain and bias terms and *D*[*n*] is the cantilever deflection. It is important to note that for this relation to hold, the transient response of the lock-in signal must be faster than the refresh rate of the discrete-time controller and is described in detail in Supplementary Note 1. The demodulated lock-in signal *L* can be applied to a discrete-time integral controller to determine the piezo modulation voltage *v*_2_ defined as follows

3 $$v_2\left[ {n + 1} \right] - v_2\left[ n \right] = K_I\left[ n \right]\left( {s - L\left[ n \right]} \right).$$

Here *K*_*I*_[*n*] is the time-varying integral gain, *s* is the controller set-point and *L* is the lock-in modulation voltage. The piezo modulation voltage from the integral controller is applied to a custom quadrature amplitude modulator (QAM) which allows for analog control of the amplitude and phase of a harmonic voltage signal. Implementation of the integral controller and design of the QAM are described in the methods and Supplementary Note 2 respectively. The output of the QAM is then applied to the piezo shown in Fig. 1a to drive a spatially constant, out-of-plane sample expansion. Note the piezo actuator used for the CL control is separate from the piezo stage used for height tracking and sample scanning.^37^ Figure 1b depicts the complex-valued, discrete-time CL controller diagram for the piezo modulation voltage which incorporates the cantilever, lock-in and integral controller relations.

To visualize the processing of these complex modulation signals, we utilize a phasor diagram to illustrate their relationships (Fig. 1c). The complex signals ${\it{\epsilon }}_1$ and ${\it{\epsilon }}_2$, representing the harmonic photo and piezo induced expansion amplitudes respectively, add as vectors in the complex plane, resulting in the total surface expansion, ${\it{\epsilon }}$. The total expansion vector is scaled and rotated by the cantilever transfer function *H*_*c*_ (or responsivity) resulting in the cantilever deflection *D* at the probe laser focus position. The reflected probe laser is converted to a voltage via a quad diode, recorded with the lock-in and processed (again scaled and rotated through *H*_*p*_ and *K*_*I*_) such that the sum of the complex expansion vectors ${\it{\epsilon }}_1$, ${\it{\epsilon }}_2$ and $\Delta {\it{\epsilon }}_2$ reduce the magnitude of the total expansion ${\it{\epsilon }}$. For stable CL operation, the piezo expansion ${\it{\epsilon }}_2$ will converge to the QCL photo-induced expansion ${\it{\epsilon }}_1$. Thus, the recorded piezo modulation voltage will be proportional to the modulation amplitude of the harmonic photo-induced expansion signal, independent of spatial and temporal cantilever responsivity variations. We note that this method can have wide applicability. Recording any heterodyne expansion signal in the manner proposed can be applied to many similar modalities including AFM-IR^41^, nano-vis^42^, and Scanning-Joule Expansion Microscopy (SJEM)^43^ with similar performance benefits.

### Optimized controller tuning for robust CL AFM-IR imaging

To assess the behavior of this design and ensure optimal performance, we can rearrange the governing equations as a complex-valued, discrete time-varying state space with the following form

4 $$\begin{array}{*{20}{l}} {x\left[ {n + 1} \right]} \hfill & = \hfill & {A\left[ n \right]x\left[ n \right] + B\left[ n \right]u\left[ n \right],} \hfill \\ {y\left[ n \right]} \hfill & = \hfill & {Cx\left[ n \right] + Du\left[ n \right].} \hfill \end{array}$$

Here *x* is the state vector, *u* is the input stimulus, and *y* is the desired output signal. *A*, *B*, *C* and *D* are the state, input, output and feedthrough matrices (time-varying, complex-valued scalars in this case) respectively. Equations (1)–(3) can be combined to determine the state equation from (4) by eliminating the lock-in and deflection signals to explicitly relate the piezo voltage and photo-induced expansion. Additionally, we will define the state-space output, *y*, to equal the piezo modulation voltage since this is the desired measureable signal. By applying these operations, we can show the state, input and output vectors of the state-space representation to be the following

5 $$\begin{array}{c} x\left[ n \right] = v_2\left[ n \right],\\ u\left[ n \right] = H_p^{ - 1}{{\epsilon }}_1\left[ n \right]+H_p^{ - 1}H_c^{ - 1} \left[n\right]\left(b-\frac{S}{\vert H_{L}\vert}\right)\\ y\left[ n \right] = v_2\left[ n \right].\end{array},$$

The state of the system and the output are both equal to the piezo voltage which (for stable operation) will converge to the input vector *u*. The input vector is, by design, proportional to the desired photo-induced expansion, ${\it{\epsilon }}_1$, but also the lock-in bias, *b*. Non-zero bias signal may result from either bias voltage in the electronics or non-local sources of signal such as photo-induced sample acoustics or non-local heating of the cantilever. The bias term serves as a source of additive noise when defining the system performance and is often assumed to have zero mean value which is valid under most sample conditions. The state-space matrices are defined as follows

6 $$\begin{array}{*{20}{l}} {A\left[ n \right] = \left( {1 - \left| {H_L} \right|K_I\left[ n \right]H_c\left[ n \right]H_p} \right),} \hfill & {B\left[ n \right] = - \left| {H_L} \right|K_I\left[ n \right]H_c\left[ n \right]H_p,} \hfill \\ {C = 1,} \hfill & {D = 0.} \hfill \end{array}$$

Without loss of generality, the system has a time-varying response as a result of both spatial and transient cantilever responsivity variations *H*_*c*_[*n*]. A time-invariant system, however, is desired as it would allow for quantitative and optimal control of the system’s stability and performance. To approximate a time-invariant response, the integral gain *K*_*I*_[*n*] needs to be adjusted such that the state-space matrices are constant with respect to time. An easy way to implement this idea is to measure the lock-in deflection voltage produced by driving the piezo with a constant voltage in order to estimate $\left| {H_L} \right|H_c\left[ n \right]H_p$ as a function of sample position. The CL measurement can then be collected on a subsequent scan with the integral gain *K*_*I*_[*n*] set as follows

7 $$K_I\left[ n \right] = K_o\left( {\left| {H_L} \right|H_c\left[ n \right]H_p} \right)^{ - 1}.$$

With this approach, the controller will function with a time-invariant response and performance (SNR, response time, etc.) set by the complex constant *K*_*o*_, whose optimal value can be determined by a time-invariant performance analysis. Assuming the matrices are constant for the measurement time, we can analytically describe the time-invariant system transfer function as follows

8 $$\begin{array}{*{20}{l}} {y\left[ n \right]} \hfill & = \hfill & {\mathop {\sum}\limits_{l = - \infty }^\infty g \left[ l \right]u\left[ {n - l} \right]} \hfill \\ {g\left[ n \right]} \hfill & = \hfill & { - \left\{ {\begin{array}{*{20}{l}} {\delta \left[ {n - 1} \right]} \hfill & {\mathrm{if}} \hfill & {Ke^{i\theta } = 1} \hfill \\ {\frac{{Ke^{i\theta }}}{{Ke^{i\theta } - 1}}\left( {\delta \left[ n \right] - \left( {1 - Ke^{i\theta }} \right)^n} \right)} \hfill & {\mathrm{if}} \hfill & {\mathrm{else}} \hfill \end{array}} \right.} \hfill \end{array}$$

The piezo voltage signal (state-space output *y*) is equal to the discrete-time convolution of the input vector *u* with the CL controller transfer function *g*[*n*] where $Ke^{i\theta }$ is the complex-valued controller gain defined as follows

9 $$\begin{array}{*{20}{l}} {K = \left| {H_L} \right|\left| {K_I} \right|\left| {H_c} \right|\left| {H_p} \right|,} \hfill \\ {\theta = \angle K_I + \angle H_c + \angle H_p.} \hfill \end{array}$$

The behavior of this system (by design) is identical to that of Euler’s method and has the following stability criteria

10 $$\left| {1 - Ke^{i\theta }} \right| \le 1.$$

**Fig. 2:**
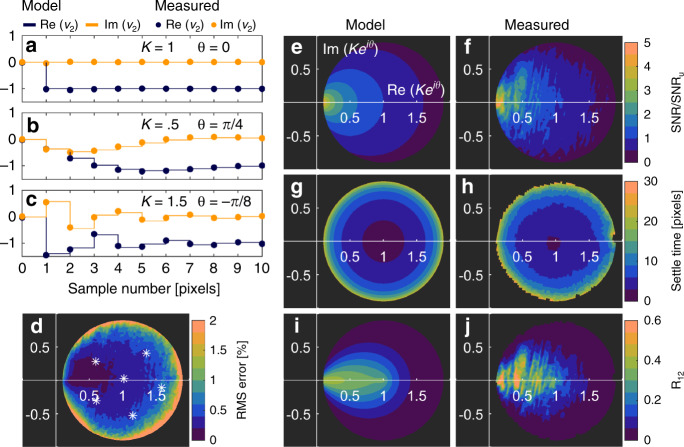
Closed-Loop AFM-IR Piezo Controller Characterization. A step voltage was applied to the piezo modulation voltage at t = 0 for 150 × 150 values of complex integral gain $\left( {{\mathrm{Re}}\left( {{{K}}_{{I}}} \right) \times {{Im}}\left( {{{K}}_{{I}}} \right)} \right)$ and then repeated eight times for calculating signal to noise ratio (SNR) as a function of the complex controller gain *Ke*^*iθ*^. **a**–**c** Complex piezo control voltage step response with model fit and measured data for select controller gain values. The controller has a minimum response of 1 iteration and converges to the opposite of the input signal when operated in the stability region of the controller gain defined as |1−*Ke*^*iθ*^| ≤ 1. **d** RMS error between the model and measured step response as a function of the controller gain. The white points indicate the data used to fit the model and measured step response. **e**, **f** Controller SNR enhancement as a function of controller gain for the model and measured data respectively. **g**, **h** Controller settle time for the model and measured data respectively. **i**, **j** Normalized Pixel Rate (NPR) for the model and measured data respectively. Optimal controller performance (maximum NPR) is achieved for any real-valued controller gain *Ke*^*iθ*^ between 0 and 1. Operation in this optimal regime can be achieved by measuring the cantilever responsivity and using that estimate to set the integral gain *K*_*I*_.

To quantify the performance of the controller, we applied a step function to the piezo for an array of 150 × 150 values of the integral gain *K*_*I*_ and fit the theoretical step response to the measured data. Figure 2a–c shows the controller step response for three values of the controller gain and Fig. 2d shows the RMS error of the fit for all values of the controller gain within the stability region. For each step response within the stability region, the piezo voltage signal approaches a value of minus one, equal and opposite of the step input as expected for destructive interference of two complex signals. Additionally, the transfer function acts as an electronic filter which affects the noise and response time of the controller output as a function of the complex controller gain $Ke^{i\theta }$. To determine the optimal controller gain, we need to determine the controller settle time and signal to noise ratio (SNR) associated with the convolution of the controller transfer function with a noisy input. The controller SNR can be described as follows

11 $${\mathrm{SNR}} = {\mathrm{SNR}}_u\sqrt {\frac{{2\cos \left( \theta \right) - K}}{K}} .$$

The SNR of the piezo control voltage (SNR) is proportional to the SNR of the input signal (SNR_*u*_) and an additional function of the gain parameters resulting from signal smoothing of the controller transfer function. Figure 2e, f shows the model and measured SNR of the piezo voltage respectively. For values of *K* approaching zero, the transfer function smooths the input signal, lowering the noise at the expense of controller response, defined by

12 $$T_s = \frac{{2\ln \left( {.1} \right)}}{{\ln \left( {K^2 - 2K\mathrm{cos}\left( \theta \right) + 1} \right)}} + 1.$$

The controller response (settle time) describes the time elapsed from application of a step input for the piezo voltage to remain within a 10% error band of the final voltage. This settle time constrains the minimum sampling rate for recording independent samples. Figure 2g, h shows the model and measured controller settle time plots respectively. The fastest response occurs in the center of the stability region, but the SNR improves near zero. The optimum performance can be determined by definition of the Normalized Pixel Rate defined as follows

13 $$R_{21}\left( {K,\theta } \right) = \frac{{n_2}}{{n_1}}\frac{{t_1}}{{T_s}}\left( {\frac{{\mathrm{SNR}}}{{\mathrm{SNR}_1}}} \right)^2.$$

This metric allows for comparing the performance of two measurements, assuming the validity of the commonly accepted square root relationship between number of samples averaged and noise. We can directly compare the performance of OL to CL (measurements 1 and 2 respectively) for an equal number of samples (*n*_1_ = *n*_2_) where every OL pixel has SNR equal to the input (SNR_1_ = SNR_*u*_) and response time equal to half a CL pixel (*t*_1_ = 5) due to the time dedicated for processing and writing the piezo output voltages. Figure 2i, j shows the model and measured normalized pixel rate plots respectively. The optimum set point for controller gain *K*_*o*_ lies anywhere on the real axis between values 0 and 1 for optimal NPR. Because there is some variability in our estimate of |*H*_*L*_|*H*_*c*_[*n*]*H*_*p*_, it is best to set the value of *K*_*o*_ equal to 0.5 in the center of the optimal NPR.

Here after, all CL data reported in this paper will incorporate this value of *K*_*o*_ applied to the gain measurement approach described above and operate at a fixed laser repetition rate. Additionally, all responsivity corrected signals (both OL and CL) will represent estimates of the input signal *u* and will be labeled as such without signal averaging or smoothing. Derivations of the equations in this section can be found in Supplementary Note 3.

### Minimizing time-varying cantilever resonance effects with CL AFM-IR

**Fig. 3:**
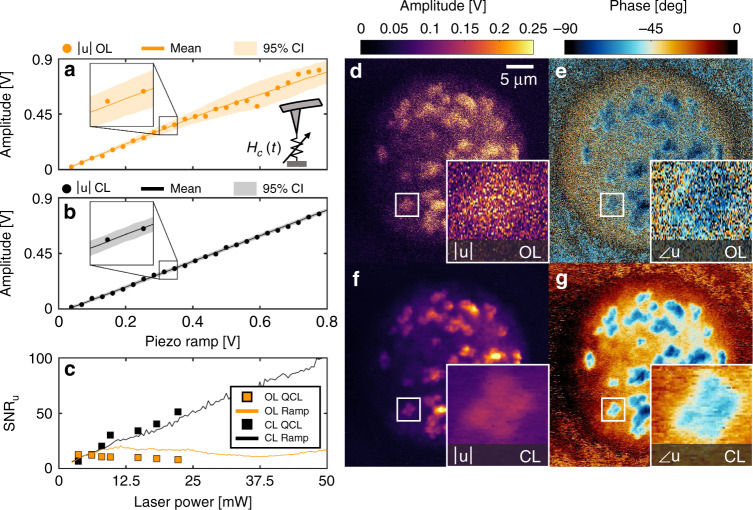
Effects of time-varying cantilever resonance in AFM-IR. **a** Amplitude of the cantilever deflection voltage demodulated at fixed 1007 kHz (6th resonance mode) resulting from driving the piezo amplitude from 0 to 1 V within 0.5 s (single scan). The recorded deflection signal was scaled by an averaged measurement of responsivity to match the closed-loop signal |*u*| while retaining the noise behavior of the raw deflection signal. Data points represent samples taken from a single representative scan plotted with mean and 95% confidence intervals (CI) of 50 repeat scans. Signal variance (noise) was observed to be constant for low signal (below. 1V) and then increases linearly with the cantilever deflection. **b** Amplitude of the closed loop (CL) responsivity corrected signal under the same conditions as (**a**). The CL signal exhibits constant noise equal the minimum noise achieve in the OL signal (i.e. at 0V from (a)). (**c**) Signal to noise ratio (SNR) for the OL and CL ramped signals from (**a**) and (**b**) respectively. SNR was also measured using the signal produced by a 400 nm SU8 film heated by a Quantum Cascade Laser (QCL) at discrete laser power levels for both OL and CL modes. The laser power was correlated to the ramp voltage (x-axis) using the relation between the CL signal and laser power. The SNR of the QCL driven signal exhibits the same behavior as the piezo ramp measurements suggesting the OL noise scales with cantilever deflection (not laser power) most likely due to time-dependent variations in the cantilever’s resonance behavior. **d**, **e** OL amplitude and phase responsivity corrected AFM-IR images of chromosomes from a metastatic breast cancer cell line (MCF10A series) collected at fixed 360 kHz (3rd resonance). **f**, **g** Same sample and conditions as (**d**) and (**e**) except operated in CL. Both OL and CL were collected with resonance-enhanced sensitivity; however, the OL signal is degraded by rapid time-variations in resonance resulting in high noise and inaccurate phase signal due to processing the ratio of noisy measurements.

The high sensitivity of current state-of-the-art RE AFM-IR has been widely reported.^39,44–47^ Many of these reports rely on the combination of tip-enhancement and cantilever resonance for high sensitivity^28,37^. Our results indicate the possibility of improved measurements by operating with resonance sensitivity while minimizing time-varying cantilever resonance effects using CL controls. Figure 3a shows the demodulated cantilever deflection signal at 1007 kHz (6th resonance) in response to a ramped modulation voltage applied to the piezo over a 0.5 s scan. This measurement was performed on a 400 nm thick SU8 film and repeated 50 times. The variance of repeat scans is constant for piezo voltages below 0.1 V but grows linearly with increasing signal. Figure 3b shows the same dataset collected while operating in CL with constant variance among repeat scans equal to the minimum value achieved in OL. To our knowledge, this is the first demonstration of multiplicative noise sources in AFM-IR and highlights the importance of CL operation to further improving AFM-IR measurements. However, the source of this variance cannot be ascertained from piezo ramp data alone. Figure 3c shows the SNR of the ramp measurement in OL and CL, as well as SNR of measurements driven by photo-induced expansion. Both the photo-induced and piezo ramp measurements show similar behavior, suggesting the increased noise observed in the OL signal is independent of the source of signal and likely originates instead from effects that amplify the signal, such as cantilever resonance. We hypothesize that this effect results from explicit time variations in cantilever resonance that, in OL operation, would produce noise proportional to the signal (regardless of the source). Because the CL signal *u* from Eq. (5) is independent of cantilever responsivity *H*_*c*_, rapid, time-dependent changes in resonance have minimal influence on the recorded signal. Further investigation into the CL signal SNR is provided in Supplementary Note 4.

The implications of minimizing resonance variability using CL controls are apparent when recording large signals. For example, Fig. 3d and e show the OL amplitude and phase Amide II absorption images of chromosomes collected at 360 kHz (3rd resonance) without signal averaging or smoothing. Figure 3f, g shows the same sample for CL operation. Time-dependent variations in cantilever resonance result in a significant increase in noise in the OL images. Further, inaccurate phase signal trending toward a −90° phase (blue) can be observed on the substrate compared to the CL dataset, which can be expected when processing the ratio of noisy measurements. Thus, conventional AFM-IR techniques and the OL approach are less effective when measuring small signals such as chromosomes over large DC signals which amplify noise. This is the factor that limits use of OL AFM-IR to small, isolated absorbers on IR compatible substrates such as gold,^33,47^ zinc selenide (ZnSe),^45^ or polymer coated ZnSe.^34^ For the same reason, our CL approach can enable these measurements on more commonly used substrates such as glass and Silicon with minimal loss of performance.

### Enabling nanoscale IR absorption measurements on arbitrary substrates

Far-field IR absorption measurements provide rich molecular information through analysis of both the shape and position of absorption bands. For example, analysis of the Amide I band shape is often used to characterize the secondary structure of proteins which has been widely demonstrated with both far-field^48^ and near-field techniques.^49,50^ Although the low noise of far-field FT-IR techniques has enabled reliable identification of peak characteristics, enabling nanoscale equivalent measurements remains a challenge due to artifacts, noise and non-linear signals. Scattering-based near field methods, for instance, rely on optimally designed cantilever probes for sufficient sensitivity; however, tip-enhancement results in a non-linear signal and can cause artifacts arising from spatial heterogeneity that can limit both chemical fidelity and mapping capabilities.^51,52^ AFM-IR offers an alternative route to record nanoscale signal linearly correlating to far-field absorption^31,53^; however, conventional methods do not provide the necessary performance to enable reliable measurements of thin materials on common nanoelectronic substrates. Here, we demonstrate the utility of the CL method by measuring IR absorption of 100 nm thick PMMA film applied to glass.

**Fig. 4:**
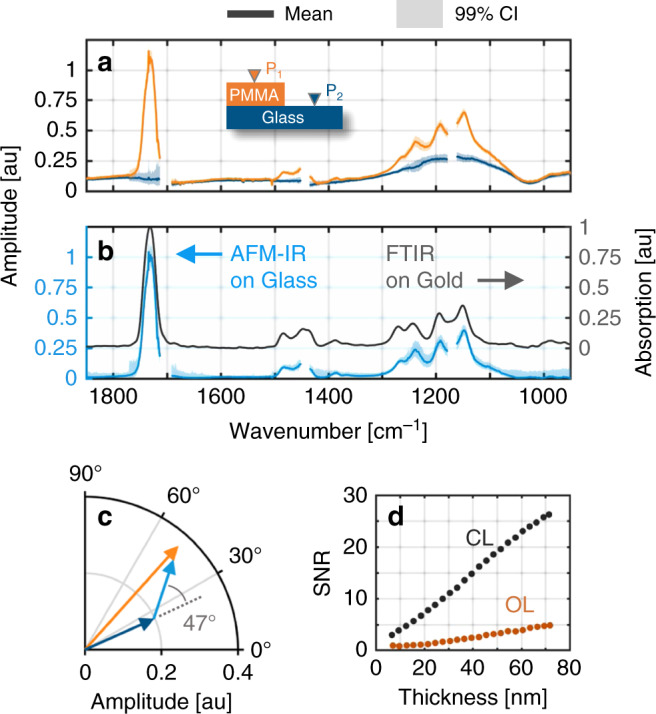
Nanoscale infrared absorption measurements on glass. **a** Infrared point spectra collected in closed-loop on 100 nm PMMA film (orange) and on glass substrate (blue). Data were omitted at QCL laser transitions due to artifacts and increased noise resulting from insufficient spot focus and low laser power. **b** Difference spectrum of PMMA and glass spectra from (**a**) compared with FT-IR reflection-absorption spectra of 100 nm PMMA film on gold. **c** Complex amplitude of point and difference spectra at 1244 cm^−1^. The PMMA signal is 47 degrees out-of-phase from the baseline which was attributed to thermal diffusion. **d** Signal to noise ratio (SNR) versus film thickness of difference spectrum signal at 1732 cm^−1^ for closed-loop and open-loop methods.

Figure 4a shows the CL spectra (mean of 100 samples) collected at the PMMA film and glass substrate locations with 99% confidence intervals for single measurements recorded at the 4 ms pixel rate of the controller. The PMMA point spectra reveal characteristic spectral features riding on a baseline signal, which resembles the glass spectra. For conventional AFM-IR, spectra collected at two separate sample locations can exhibit different scaling due to spatially varying responsivity effects and is often normalized in an ad hoc manner. Moreover, because the expansion signals are harmonic, the substrate and sample contributions can exhibit interference effects which cannot be corrected using conventional RE or PTIR amplitude measurements alone. Only responsivity corrected measurements provide properly scaled, complex-valued signal allowing for reliable correction of the observed substrate baseline signal. Figure 4b shows the difference spectra of the PMMA and glass sample locations compared to FT-IR reflection-absorption spectra of 100 nm PMMA film on gold. The difference spectrum shows good agreement with bulk FT-IR measurements, enabling accurate chemical analysis of nanomaterials on common substrates.

The CL method enables accurate detection of the complex-valued photo-induced expansion, which is paramount to ensure reliable absorption measurements of nanomaterials. Figure 4c shows the complex phasor plot of the point and difference spectra for 1244 cm^−1^ revealing a 47° phase shift between difference and baseline signals. Phase shifted signal can occur as the result of a number of effects such as thermal diffusion of the photo-induced heat. Recording only the harmonic amplitude typical of conventional AFM-IR and processing the difference, for example, would result in approximately 32% error at the 1244 cm^−1^ peak. Moreover, in theory, both OL and CL responsivity corrected measurements should result in the same complex-valued signals; however, processing the ratio of noisy OL measurements cannot only result in heightened levels of noise but also inaccurate signal, which was observed previously with the chromosome sample. Figure 4d shows the SNR of the 1732 cm^−1^ absorption peak for CL and OL methods for PMMA thickness down to 10  nm demonstrating ~5× improvement in noise. Thus, the CL method provides a complete, complex-valued measurement with minimal noise for accurate detection of nanothin materials on arbitrary substrates.

### Mapping molecular information of nanomaterials

**Fig. 5:**
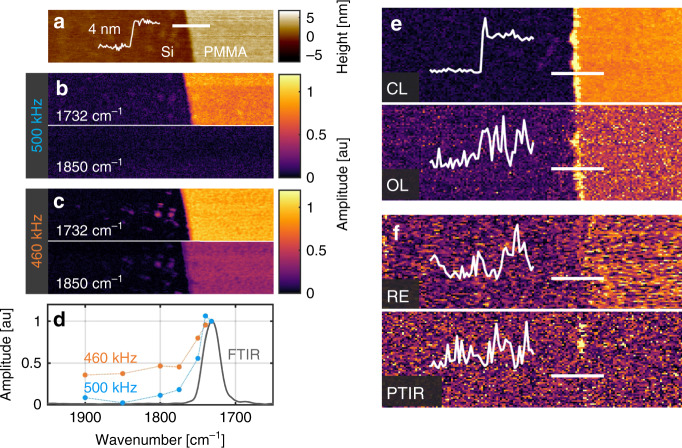
Mapping Molecular Information of Nanothin Materials on Silicon. **a** Height map of a 4 nm thick PMMA film on Silicon. **b** Closed-loop (CL) baseline corrected infrared maps of 1732 and 1850 cm^−1^ of the 4 nm film collected off resonance at 500 kHz. **c** Same measurement as (**b**) collected at 460 kHz near the cantilever’s 3rd resonance mode. **d** Magnitude of the PMMA film signal extracted from CL hyperspectral images compared to FTIR. CL image contrast recorded near cantilever resonance had increased sensitivity (i.e., lower substrate noise) but also additional signal uncorrelated with the local PMMA absorption which was attributed to photo-induced sample acoustics. **e** Comparison of CL and OL maps at 1732 cm^−1^. Line profiles reveal improved signal to noise ratio (SNR) for CL operation. The color scale for the OL measurement was scaled by a factor of 2 due to the increase noise. **f** Same maps as (**e**) using previous state-of-the-art Resonance Enhanced (RE) and Photo Thermal Induced Resonance (PTIR) methods. For RE measurements, the 3rd resonance mode was tracked using the commercial Nano-IR2 software. Overall this slowed acquisition time down by a factor of 2.5 in addition to worse performance when compare to CL. For PTIR measurements, coaveraging was set to produce a pixel rate equivalent to the CL and OL measurements.

A number of reports demonstrate high-sensitivity AFM-IR imaging of nanothin materials such as 2D materials,^34,35^ Self-assembled monolayers (SAM)^33^ and isolated proteins^47^; however, accurate AFM-IR absorption measurements on arbitrary substrates is often limited by non-local signals, non-chemical effects (responsivity) and noise. The CL method is designed to address these challenges. To demonstrate its capabilities, a 4 nm thick PMMA film was applied to Silicon as shown in the height map of Fig. 5a. Figure 5b shows the 1732 and 1850 cm^−1^ baseline corrected CL absorption maps of the same region collected at 500 kHz (off resonance). The CL measurements reveal accurate maps of the localized PMMA absorption with little contribution from other non-chemical effects. Collecting this same data at 460 kHz (near 3rd resonance) shown in Fig. 5c results in improved sensitivity (based on reduced substrate noise); however, the 1850 cm^−1^ map reveals relatively high signal on the PMMA film. Figure 4h shows the PMMA film signal extracted from CL hyperspectral images compared to FT-IR absorption normalized by the 1732 cm^−1^ peak. Data collected at 500 kHz (off resonance) shows good agreement with FTIR absorption data and tends toward the noise floor at 1900 cm^−1^; however, the 460 kHz data (near 3rd resonance) seems to plateau at some other limiting value. We suspect photo-induced sample acoustics^30^ or other non-local sources of cantilever heating^37,41,54^ are driving the cantilever signal producing a non-zero bias signal that is further amplified under resonance conditions. According to the CL signal defined in Eq. 5, a constant bias value *b* can become encoded spatially through variations in cantilever responsivity $H_c^{ - 1}b$ resulting in non-chemical contrast even after baseline correction. This is just one of a number of effects which are now detectable with the improvements in noise and accuracy of the CL method. Further investigation is required to understand the true nature of these signals to enable accurate chemical measurement with the high sensitivity of cantilever resonance. Regardless, even operating off resonance, the CL method provides accurate chemical signal with optimal noise compared to previous state-of-the-art deflection-based measurements.

Figure 5e shows a comparison of CL and OL 1732 cm^−1^ absorption maps collected at 500 kHz (off resonance). The line profiles reveal significantly reduced noise while operating in CL consistent with previous ramp and chromosome measurements. Figure 5f shows the same measurements collected using prior RE and PTIR deflection amplitude measurements revealing high levels of noise and questionable chemical contrast in addition to reduced pixel rate to allow for resonance tracking and signal averaging respectively. Parameters of RE and PTIR measurements are provided in the methods section. The 4 nm film is easily resolved and its signal can be quantified in the CL approach. Notably, IUPAC guidelines typically recommend a signal 10-fold greater than noise to quantify results. In this case, only the CL method provides the necessary measurement performance to quantify spectra and can be a reliable method for sub-10 nm films. For example, we note that the slight increase in the intensity of the edge of the PMMA film is ~1 nm and can be easily measured with the noise floor seen on the ordinary silicon substrate. In terms of measurement capability, thus, this work moves AFM-IR sensitivity to the realm of organic monolayers of widespread importance such as supported lipid bi-layers and self-assembled alkanethiols that are typically 1–5 nm thick. While this work has analyzed some of the more apparent sources of noise and presents a means to decrease the same, the high quality data now also allow investigation into further sources of noise and can potentially result in greater improvements to the quality of recorded data. We note that the results also illustrate the general applicability of our approach to conventional AFM-IR instrumentation. With the CL approach, existing AFM-IR users can rapidly implement the described improvement and obtain more consistent and accurate data. Not only is the CL approach highly compatible with existing instrumentation, its deployment can also make the conduct of experiments more user-friendly to make nanoscale chemical measurements more accessible.

## Discussion

Near field spectroscopy methods often require tip- or sample-induced signal enhancement that permit sensitive measurements but also make signal and noise difficult to predict and optimize. Consequently, AFM-IR offers the potential for high sensitivity and fidelity nanoscale chemical imaging but has been limited to small, isolated samples on specific substrates. Here, we first demonstrate that the performance of AFM-IR is limited by the effects of time-varying cantilever resonance that result in a significant increase in noise at large cantilever deflection, especially for samples that produce a large DC bias signal. We then utilized this insight to devise a CL AFM-IR approach. As opposed to conventional AFM measurements that emphasize larger cantilever deflections, the CL approach maintains near-zero cantilever deflection while measuring an applied signal to a subsample piezo instead using feedback control. This CL control strategy results in a regime where noise and saturation effects are minimal, enabling high sensitivity IR absorption measurements on arbitrary substrates. We provide a complete analysis of the proposed controls for robust, optimal performance. We then implemented the concept on a standard commercial AFM-IR instrument and characterized the advancement. The improved sensitivity and reliable phase signal unique to the CL method are shown to improve data collection and processing to enable nanoscale composition mapping on common substrates such as Silicon and glass. This advance augments AFM-IR to provide metrology capabilities to a wide range of fields in need of reliable nanoscale composition imaging such as high frequency nanoelectronics^6,7^, NEMS and MEMS^8^, and photonics^9^.

## Methods

### CL hardware and implementation

All AFM-IR data were collected using a modified Nano-IR2 from Anasys Instruments Corporation (now Bruker Corporation) using a standard Anasys contact mode probe (PN PR-EX-nIR2–10). The CL integral controller was implemented using a hardware-timed single point I/O described in the Supplementary Note 1. The high-speed deflection voltage of the AFM was accessed via the J12 SMA connector on the back panel of the Nano-IR2 and applied to a SR844 lock-in amplifier. The demodulated X and Y channels of the lock-in (with 10 V output) were fed to analog input channels of a PCIe-6361 DAQ (set with 10V dynamic range) and processed with the gain-scheduled integral controller to determine the piezo modulation voltages. The piezo voltages were sent to the analog output channels on the DAQ and applied to the QAM circuit which is described in Supplementary Note 2. The CL and OL controller signals were mapped to the sample position by counting the pulses of a 20 MHz clock synchronized to start at the beginning of every trace and retrace scan using the Nano-IR2 scan line trigger.

### Data collection and processing

Resonance enhanced AFM-IR measurements were conducted using the commercial Nano-IR2 software with a 20 kHz scan range, 0.01 s scan time and 10 points per scan for tracking 3rd resonance mode. Conventional PTIR measurements were collected by pulsing the laser at 1 kHz while measuring the peak deflection voltage which was band pass filtered at the 2nd resonance mode where signal was highest. Averaging of the PTIR signal was adjusted between 4 and 32 scans to maintain CL-equivalent pixel rates, but produced little improvement in SNR. All data were reported with no additional post-processing or smoothing.

### Sample preparation

The 100 nm PMMA film was applied to glass via spin coating at 3000 rpm for 80 s using PMMA photoresist from MicroChem Corporation (PN 950PMMA A2). The film was scratched and heating to 160 °C overnight to produce a polymer ramp for thickness measurements. The 4 nm PMMA film was applied to Silicon via spin coating at 3000 rpm using a 16% w/w dilution of the PMMA A2 photoresist in Anisole.

MCF10A normal human mammary epithelial cell line was obtained from the American Type Culture Collection (ATCC) and cultured using the standard protocol prescribed by ATCC. The cells were grown to a confluency of 40–70% to ensure that they were actively dividing. The cells were then treated with colcemid to achieve a final concentration of 0.5 μg/ml (this can range between 0.5 and 1.0). These cells were incubated for 10 h at 37 °C. The cell flask was tapped and the colcemid solution was collected in a conical tube. The flask was washed with 10 ml sterile PBS, tapped and the solution was collected into a conical tube. This was subsequently centrifuged for 5 min at 1000 rpm. When cells were adherent to the flask potentially resulting in insufficient cell yield, a quick rinse (~1 min) with warm trypsin was done. Three milliliters of warm trypsin was added to the flask and incubated for 1 min. The floating cells were checked and 7 ml of fresh warmed (37 °C) media was added. The flask was tapped, and the media and trypsin were collected in a conical tube. All tubes (can be mixed into one) were centrifuged for 5 min at 1000 rpm. The supernatant was aspirated, and the pellet was gently resuspended in 5 ml of 0.4% KCL (hypotonic solution). The suspension was incubated for 25 min at 37 °C. Next, 5 ml of fixative (3:1 methanol-acetic acid solution) was added and kept for 10 min The tube was centrifuged at 1000 rpm for 10 min The pellet was resuspended in 10 ml of fixative again for 10 min and this step was repeated three times. To prepare the sample, gold coated Si wafers were first sterilized and snap frozen using liquid nitrogen. Ten microliters of cell suspension was dropped to the frozen substrate from a height of about 60 cm (this height was optimized for our system and the desired yield). This caused the swollen nuclei to break and the chromosomes to spread out so that they can be analyzed individually. The samples were placed in a vacuum oven to dry.

## Supplementary information

Supplementary Information

## Data Availability

All data generated or analyzed during this study are included in this published article (and its supplementary information files). The datasets generated during and/or analyzed during the current study are available from the corresponding author on reasonable request.
