# Supplementary material for: Closed-loop atomic force microscopy-infrared spectroscopic imaging for nanoscale molecular characterization
Source: Nat Commun. 2020 Jun 26;11:3225. doi: 10.1038/s41467-020-17043-5 (PMC7320136; doi:10.1038/s41467-020-17043-5)
Supplement: Supplementary file 1 — Supplementary Information [file 41467_2020_17043_MOESM1_ESM.pdf]

Supplementary Information:
Closed-Loop Atomic Force Microscopy-Infrared Spectroscopic Imaging for Nanoscale Molecular Characterization

Seth Kenkel†§, Shachi Mittal† and Rohit Bhargava†§‡*

†Beckman Institute for Advanced Science and Technology, University of Illinois at Urbana Champaign, Urbana, IL 61801, USA
§Department of Mechanical Engineering, University of Illinois at Urbana Champaign, Urbana, IL 61801, USA
‡Cancer Center at Illinois and the Departments Chemical and Biomolecular Engineering, Bioengineering, Electrical and Computer Engineering, and Chemistry, University of Illinois at Urbana-Champaign, Urbana, IL 61801, USA

*rxb@illinois.edu

TABLE OF CONTENTS

Supplementary Note 1: Controller Implementation and Transient Behavior

Supplementary Note 2: Piezo Driving Circuit

Supplementary Note 3: Time-Invariant State-Space Model

Supplementary Note 4: Input Signal to Noise Ratio Model

Supplementary Figure 1: Controller Implementation and Transient Behavior

Supplementary Figure 2: Piezo Driver Circuit

Supplementary Figure 3: Closed-Loop Signal to Noise Ratio Plot

SUPPLEMENTARY NOTE 1: CONTROLLER IMPLEMENTATION AND TRANSIENT BEHAVIOR

The integral controller for processing the Lock-In signal was implemented with a hardware-timed I/O in Labview using a PCIe-6361 DAQ device from National Instruments. The labview code reads the lock-in modulation voltages and stage position on every clock rising edge spaced every $.5\tau_c$ then discards samples at half-integral time points ($.5\tau_c$, $1.5\tau_c$, $etc..$) as shown in Supplementary Figure 1a. The half integral clock pulses are used to write the two piezo modulation voltages to the DAQ analog output (AO). This method of hardware timing ensures minimal jitter between read and write operations with the available electronics. The loop time constant τ_c is set to 4 ms to allow sufficient time for processing the data (a restriction of hardware-timed I/O). Implementing the scheme with an FPGA would allow for precise, hardware synchronized processing at much faster speeds (50 ns). The AO piezo modulations voltages v_2 are wired to a custom built piezo driver circuit shown in Supplementary Figure 1b. The details of the piezo driver circuit are described in section S2. To describe a discrete-time theory using continuous time signals, we must ensure the system response (lock-in, cantilever, etc.) is sufficiently faster than the update rate of the controller. Supplementary Figure 1c shows the transient response of the deflection signal using a step input into the piezo. This demonstrates that the system response is limited only by the time-constant of the lock-in down to 100 µs. We also need to constrain the response of the lock-in be less than $.5\tau_c$ as shown in Supplementary Figure 1a to ensure the validity of the discrete-time math. For all data collected here, our lock-in time constant was set to 300 µs resulting in a settle time of about 1.2 ms.

SUPPLEMENTARY NOTE 2: PIEZO DRIVING CIRCUIT

The Quadrature Amplitude Modulator (QAM) was implemented using two 2Vpp 50% duty cycle square wave carrier signals (0 and 90 degree phase shift) generated in the DAQ with two retriggered counters synced to the TTL trigger which drives the Quantum Cascade Laser (QCL). These square wave signals were applied to C1 and C2 pins of Supplementary Figure 2a and depicted in the inset. Supplementary Figure 2b shows the measured QAM voltage output for select values of piezo voltage magnitude $|v_2|$. Supplementary Figure 2c shows the measured QAM voltage output for select values of piezo voltage phase $\angle v_2$. This circuit allows for analog voltage control of the amplitude and phase of a harmonic voltage. Although the precise signals produced by the piezo and QCL are periodic (not harmonic), the 1st harmonic is the only portion extracted using the lock-in and processed.

SUPPLEMENTARY NOTE 3: TIME-INVARIANT STATE-SPACE MODEL

Defining the closed-loop controller performance analytically requires solving for the time-invariant controller transfer function using the state-space representation from the paper. We will use the following unilateral z-transform definition.

$$F(z) = \sum_{n=0}^{\infty} f[n] z^{-n} \tag{1}$$

Here, $f[n]$ is any discrete-time function and $F(z)$ is its z-domain equivalent. Using the discrete-time-invariant state-space matrices, the controller transfer function in z-domain can be defined as follows

$$G(z) = B(z - A)^{-1} = -\left(\frac{z-1}{Ke^{i\theta}} + 1\right)^{-1} \tag{2}$$

The time-domain solution is trivially solved by applying the appropriate inverse z-transform to this equations. Moreover, the z-domain form contains information about the performance of the

controller such as stability which defines the necessary conditions for convergence of the signal. The controller is stable (and causal) if all the poles of the transfer function are contained in the unit circle in the complex plane. The poles of the transfer function are defined as follows

$$z_p = 1 - Ke^{i\theta} \tag{3}$$

Thus, the controller output will converge (or is stable) provided the controller gain is set within a shifted unit circle as stated in the main text. The step response of the system reported in the main text can be derived as the product of the transfer function and step input z-transform as follows

$$S(z) = G(z)(1 - z^{-1})^{-1} \tag{4}$$

The n-domain step response can be determined with the method of partial fractions and inverse z-transforms or by use of Mathematica or Matlab symbolic math resulting in the following

$$s[n] = \begin{cases} \delta[n] - 1 & Ke^{i\theta} = 1 \\ \left(1 - Ke^{i\theta}\right)^n - 1 & else \end{cases} \tag{5}$$

This is the step response used for characterization in the main text. The settle time of the controller can be defined as the time for the step response to be within some designated error band of unity. The settle time is a positive, real number which satisfies the following

$$|s[T_s - 1] + 1| = e \tag{6}$$

This relation describes a continuous bound to the step response shifted to approach 0 at infinite time to allow for defining the error band e. The definition is also shifted in time such that the minimum response is a single pixel. Using the n-domain definition of the step response, the bound can be written as follows

$$|s[n] + 1| = (K^2 - 2Kcos(\theta) + 1)^{\frac{n}{2}} \tag{7}$$

This result can be applied to the settle time definition with an error bound $e = .1$ to match the definition in the main text.

A single sampled value of the state-space output (or any random, complex signal) can be described as the sum of expectation value of y and a zero-mean, random complex-valued signal for the noise component. The most appropriate definition of SNR of such a signal is the magnitude of its expected value divided by Root Mean Square (RMS) average of the noise magnitude as follows.

$$SNR = \frac{|E\{y\}|}{\sqrt{E\{|y - E[y]|^2\}}} = \frac{|E\{y\}|}{\sqrt{Var\{y\}}} \tag{8}$$

The RMS average is simply the definition of the variance of the complex-valued output. The challenge is to define this in terms of the input signal using the convolution properties of the transfer function. By applying the convolution definition of the transfer function to the output and commuting operations, we can define the following

$$|E\{y[n]\}| = \left|\sum_{l=0}^{\infty} g[l]E\{u[n - l]\}\right| \tag{9}$$

Here, we will assume the input u to have a constant expectation value for all time as is the case for the measurement of a uniform polymer film used for characterization or stationary operation. Thus, the expectation value of input is a constant and can move outside the sum. By definition, the sum of $g[l]$ is equal to negative one allowing for the following result

$$|E\{y\}| = |E\{u\}| \tag{10}$$

To complete the analysis of SNR, we need a similar relation for variance. The variance of a complex-valued random variable can be split into sum of the variance of the real and imaginary terms. Applying this operation, using the convolution definition of the output and commuting operations; we can show the following

$$Var\{y\} = \sum_{l=0}^{\infty} |g[l]|^2 Var\{u[n-l]\} \tag{11}$$

As previously used, we will assume the input u has the same statistics (in this case variance) for all time points. Thus, the variance of the input is a constant and can move outside the sum resulting in the following

$$Var\{y\} = \frac{K^2}{2Kcos(\theta) - K^2} Var\{u\} \tag{12}$$

Applying this definition and supplementary equations (10) and (8) results in the SNR relation from the main text.

SUPPLEMENTARY NOTE 4: INPUT SIGNAL TO NOISE RATIO MODEL

The approach presented in the paper ensures the controller gain is maintained at a value of .5. As a result, the SNR is equal to $\sqrt{3}$ times the SNR of the input signal u. The SNR of the input signal can be written as follows

$$SNR_u = \frac{|E[\epsilon_1]|}{\sqrt{Var\left[\epsilon_1 + H_c^{-1}\left(b - \frac{s}{|H_L|}\right)\right]}} \tag{13}$$

Here, we combined the definitions of SNR and the input signal u from the paper assuming H_p is constant and the expected value of $b - \frac{s}{|H_L|}$ is zero. The SNR of the input signal will depend on the sample expansion ϵ_1; however, we cannot measure the expansion directly so the definition

in this form is not useful. Instead, recording the deflection voltage in open-loop allows us to estimate the expansion scaled by the transfer function. Thus, we will rearrange this equation by multiplying the top and bottom by $|E[|H_L|H_c]|$ resulting in the following

$$SNR_u = \frac{|E[L]|}{\sqrt{E[|E[|H_L|H_c]\epsilon_1 + E[R]R^{-1}(|H_L|b - s) - E[L]|^2]}} \tag{14}$$

Here, we've defined the expected value of the open-loop deflection $E[L]$ equal to the term $E[|H_L|H_c\epsilon_1]$ and defined the cantilever responsivity R by grouping$|H_L|H_cH_p$ appropriately. Now we need to apply noise models for expansion and bias voltage as follows.

$$\begin{gathered} \epsilon_1 = E[\epsilon_1](1 + N_3) \\ (|H_L|b - s) = N_1 \end{gathered} \tag{15}$$

The terms $N_3 \sim \mathcal{N}_C(0, n_3^2)$ and $N_1 \sim \mathcal{N}_C\left(0, \frac{n_1^2}{\tau}\right)$ are circularly symmetric normal distributed random variables representing fluctuations in the expansion signal (due to factors such as laser pulse to pulse variation) and additive noise sources (such as electronic or thermal mechanical noise) respectively. Applying this definition to supplementary equation (14) results in the following

$$SNR_u = \frac{|E[L]|}{\sqrt{E[|E[L]N_3 + E[R]R^{-1}N_1|^2]}} \tag{16}$$

Expanding the bottom and dividing by $|E[L]|$ will result in the three noise terms shown in Supplementary Figure 3a. The noise parameter n_1 representing additive noise sources is a function of frequency due to thermal mechanical noise (pink noise) effects and is relatively flat (white noise) for higher frequencies. The noise of the lock-in deflection voltage with the cantilever engaged and the QCL laser off is shown in Supplementary Figure 3b. The noise parameter n_3 accounts for noise resulting from variance in the expansion which could result from laser shot noise or pulse variations in the IR laser due to other sources such as pointing stability and is

assumed to be constant. The remaining noise term $n_2(f)$ is a function of repetition rate due to the complicated dependence on the statistical behavior of the cantilever's responsivity R. An exact understanding of this term is unknown, but has been observed to exhibit worse performance near resonance due to the time-varying behavior of responsivity. Regardless, the effects of this variance in closed-loop is far weaker than operating in open-loop as shown in the inset of Supplementary Figure 3a. Reduced SNR for large deflection signal shown by the blue arrow of Supplementary Figure 3a cannot be accounted for with this analysis since the three proposed noise terms at most grow proportionally with deflection.

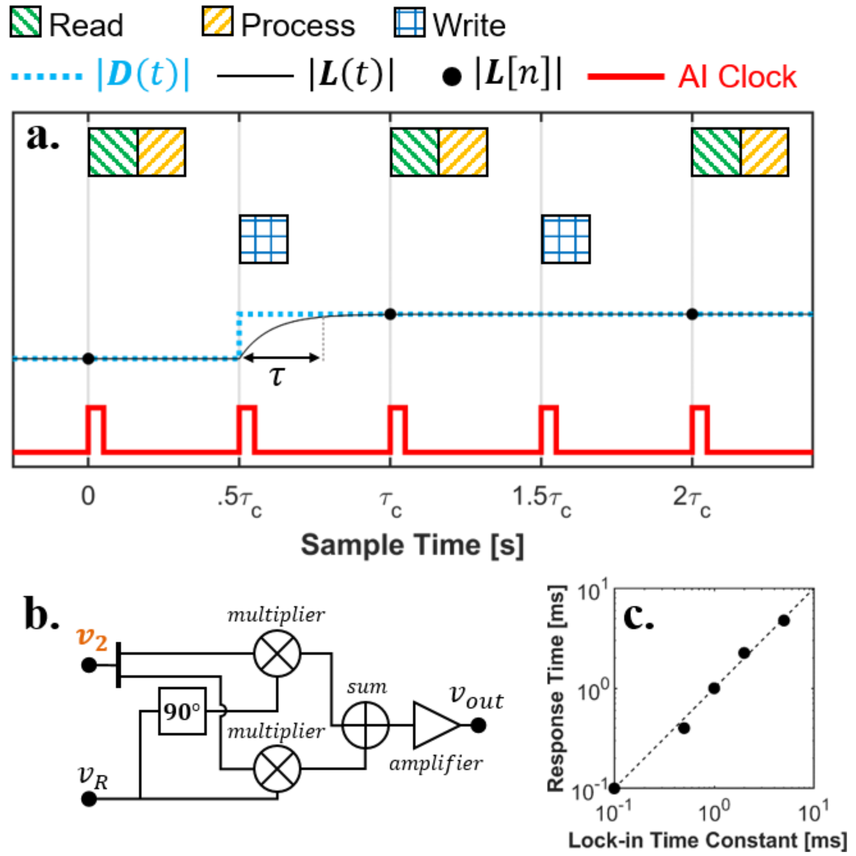

Supplementary Figure 1: Controller Implementation and Transient Behavior. (a) Hardware timed I/O diagram showing the continuous time lock-in signal $L(t)$ sampled on the rising edge of the Analog Input (AI) clock pulse every controller loop constant τ_c. The sampled, dual lock-in X and Y voltages are processed via the integral controller to produce two analog output voltages which are written to the DAQ Analog Output (AO) at hardware-timed half steps between AI read operations. The output voltages are wired to the piezo driver circuit at v_2 shown in (b) and are used to modulate a 0 and 90 degree shifted periodic (sine) reference voltage v_R thus allowing for analog control of the amplitude and phase of a harmonic voltage used to drive the piezo actuator. The reference voltage v_R and its 90 degree shifted signal are produced with the DAQ and together with the piezo driving circuit form a Quadrature Amplitude Modulator (QAM) (c) Lock-in signal response versus lock-in time constant showing a linear trend down to 100µs which suggests any transient behavior of the piezo and the cantilever are negligible for $\tau_c = 4ms$. An example perturbation of v_2 is depicted in (a) at $.5\tau_c$ which results (ideally) in an instantaneous change in the high speed, cantilever deflection signal $D(t)$. The lock-in response time τ must be set less than or equal $.5\tau_c$ in order to assume the sampled lock-in and deflection signals are identical definitions in the discrete-time description (i.e. $L[n] \propto D[n]$).

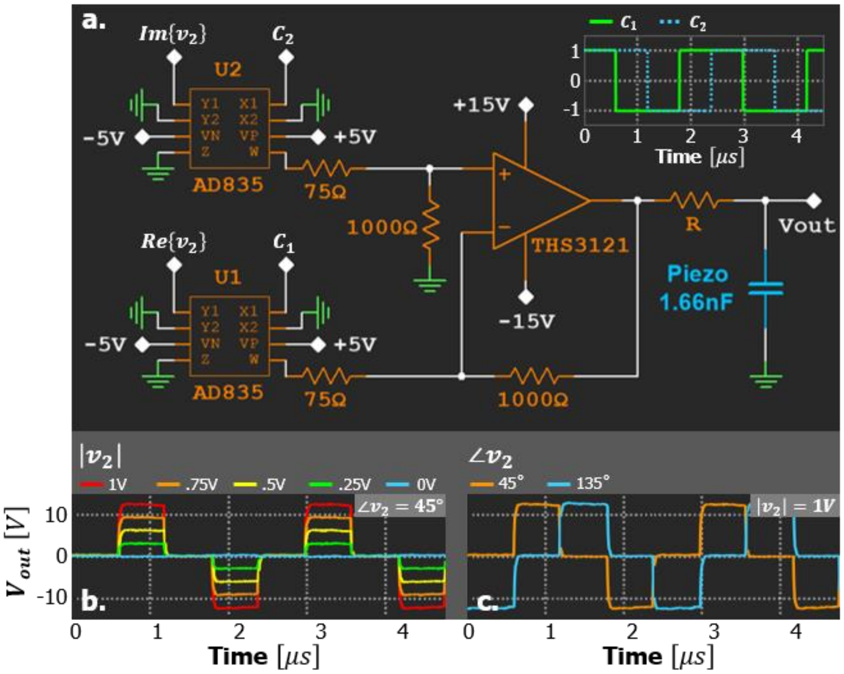

Supplementary Figure 2: Piezo Driver Circuit. (a) Piezo driver circuit schematic with two AD835 4-quadrant multipliers and one THS3121 operation amplifier. The multipliers have unity gain with 1V limits on the input pins which are connected to the in-phase and quadrature carrier signals C_1 and C_2 as well as the piezo modulation control voltage $\mathrm{Im}\{v_2\}$ and $\mathrm{Re}\{v_2\}$. The modulated signals are applied to a differential op amp with gain of ~13 resulting in a maximum of 26Vpp output voltage across the piezo. The piezo actuator was PRYY+0107 from Physik Instrumente (PI) and had a measured capacitance of 1.66nF. The series resistance R was nominally 0 Ohm and is increased to match the AFM-IR expansion signal. The bandwidth of the circuit was ~10MHz limited by the capacitive load of the piezo. The inset shows the two carrier signals which were generated and synced with the laser using the DAQ device. (b) Output voltage of the piezo driver circuit at select piezo modulation voltages. (c) Output voltage of the piezo driver circuit at two phase angles of the piezo modulation voltage.

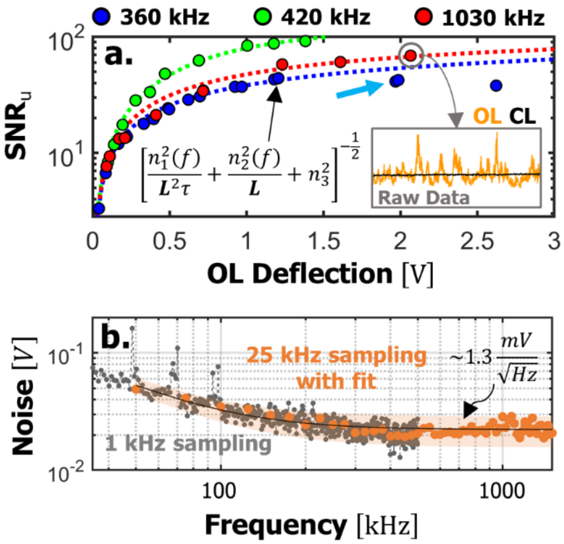

Supplementary Figure 3: Signal to Noise Ratio (SNR) of Closed-Loop (CL) AFM-IR. (a) The SNR of the CL signal increases linearly for small deflection with noise n_1 equal to additive noise sources such as electronic noise (white) or thermal mechanical noise (pink) depending on frequency. For large deflection, multiplicative noise sources dominate. These noise sources include but are not limited to QCL pulsing variations, QCL shot noise and time-varying resonance effects. The inset shows a time sampled signal for CL versus OL methods at 1030 kHz (near 6th resonance) demonstrating improved performance using CL method. OL data was scaled by an averaged measurement of responsivity to match the closed-loop signal $|u|$ while retaining the noise behavior of the raw deflection signal similar to the procedure from figure 3 of the main text. The CL signal at 360 kHz (near 3rd resonance) exhibits non-linear increase in noise attributed to time-varying resonance effects indicated by the blue arrow. (b) Lock-in deflection noise with cantilever engaged and QCL laser off. The sensitivity and time constant settings equal those used in the paper. Thermal mechanical noise excites cantilever resonance only below the 2nd resonance mode near 200 kHz and approaches a flat profile (white noise) equal to 1.3 $\frac{mV}{\sqrt{Hz}}$ for pixel rate of 4 ms.
